# Cardiocutaneous syndrome is caused by aggregation of iASPP mutants

**DOI:** 10.1038/s41420-024-02265-z

**Published:** 2024-12-18

**Authors:** Rebecca Lotz, Christian Osterburg, Birgit Schäfer, Xin Lu, Volker Dötsch

**Affiliations:** 1 Institute of Biophysical Chemistry and Center for Biomolecular Magnetic Resonance, Goethe University, Frankfurt, Germany; 2 Ludwig Institute for Cancer Research, Nuffield Department of Clinical Medicine, University of Oxford, Oxford, OX3 7DQ UK

**Keywords:** Protein aggregation, Chaperones

## Abstract

The ASPP (apoptosis-stimulating protein of p53) family of proteins is involved in many cellular interactions and is starting to emerge as a major scaffolding hub for numerous proteins involved in cancer biology, inflammation and cellular integrity. It consists of the three members ASPP1, ASPP2 and iASPP which are best known for modulating the apoptotic function of p53, thereby directing cell fate decision. Germline mutations in iASPP have been shown to cause cardiocutaneous syndromes, a combination of heart and skin defects usually leading to death before the age of five. Mutations in iASPP causing these syndromes do not cluster in hot spots but are distributed throughout the protein. To understand the molecular mechanism(s) of how mutations in iASPP cause the development of cardiocutaneous syndromes we analysed the stability and solubility of iASPP mutants, characterized their interaction with chaperones and investigated their influence on NF-ĸB activity. Here we show that three different mechanisms are responsible for loss of function of iASPP: loss of the complete C-terminal domain, mutations resulting in increased auto-inhibition and aggregation due to destabilization of the C-terminal domain. In contrast to these germline mutations causing cardiocutaneous syndromes, missense mutations found in cancer do not result in aggregation.

## Introduction

Cardiocutaneous syndromes are rare diseases that are associated with palmoplantar keratoderma, woolly hair and cardiomyopathy, often terminating in heart failure or sudden cardiac death. Two well-known examples falling under this term are Naxos disease and Carvajal syndrome, both inherited in an autosomal recessive manner and caused by germline mutations in cell adhesion proteins including plakoglobin and desmoplakin [1]. Since the maintenance of cellular integrity is essential for tissues under high mechanical stress, particularly, epidermis and myocardium are severely affected.

Similar phenotypes have been observed in mutant woe2 and wa3 mice and Poll Hereford cattle. However, instead of desmosome mutations, sequencing revealed a germline mutation in the PPP1R13L gene [2–4]. PPP1R13L encodes for iASPP, a member of the ASPP family which is best known for inhibiting p53 activity [5]. Structurally, iASPP consists of a largely unfolded N-terminus followed by a C-terminal domain (CTD), which comprises four ankyrin repeats connected to an SH3 domain. This C-terminal domain serves as a hub for a variety of binding partners, including p65 and members of the AP-1 family, implicating iASPP in inflammatory processes. Interaction of iASPP with both transcription factors inhibits their transcriptional activity [6–8] and consequently, iASPP depletion leads to an upregulation of inflammation-associated genes in mice. In addition, iASPP binds to desmoplakin at cell-cell junctions, keeping them in an insoluble, desphosphorlyated state, thereby maintaining desmosomal integrity [9]. Intriguingly, cell-specific knockout of iASPP in murine cardiomyocytes and keratinocytes results in desmosome destabilization and this alone is sufficient to induce the respective cardiocutaneous phenotype in the mouse model [10].

In recent years, more than 20 human patients have been diagnosed with iASPP mutations [11–16]. The majority of these patients are members of consanguineous families and present symptoms such as dilated or arrhythmic cardiomyopathy, skin lesions, woolly hair or vision impairment. Without receiving heart transplantation, the respective patients typically succumb to this condition before reaching the age of five years. iASPP germline mutations do not cluster in specific hotspots but are instead distributed across the entire protein, affecting both the N-terminus and the CTD. Since most of them induce frameshifts, premature termination or alterations within the C-terminal fold, it is likely that these mutations result in non-functional proteins, suggesting that the general mechanism is a loss of function; however, the precise underlying molecular mechanism remains to be fully characterized.

In this study, we aimed to investigate the thermodynamic stability of wild-type (WT) iASPP and its mutants. Our findings reveal that the stability of iASPP is reliant on its interaction with other binding partners and that mutations affect its stability, leading to protein aggregation. This insight sheds light on the molecular consequences of iASPP mutations in the germline and provides valuable information for understanding the pathogenesis of the associated disorder. Interestingly, spontaneous iASPP mutations identified in cancer patients do not show an aggregation phenotype suggesting that a potential role of mutant iASPP in tumorigenesis is based on different molecular mechanisms than the aggregation inducing germline mutations in cardiocutaneous syndromes.

## Results

### iASPP mutations found in cardiomyopathy patients are prone to aggregation

**Fig. 1:**
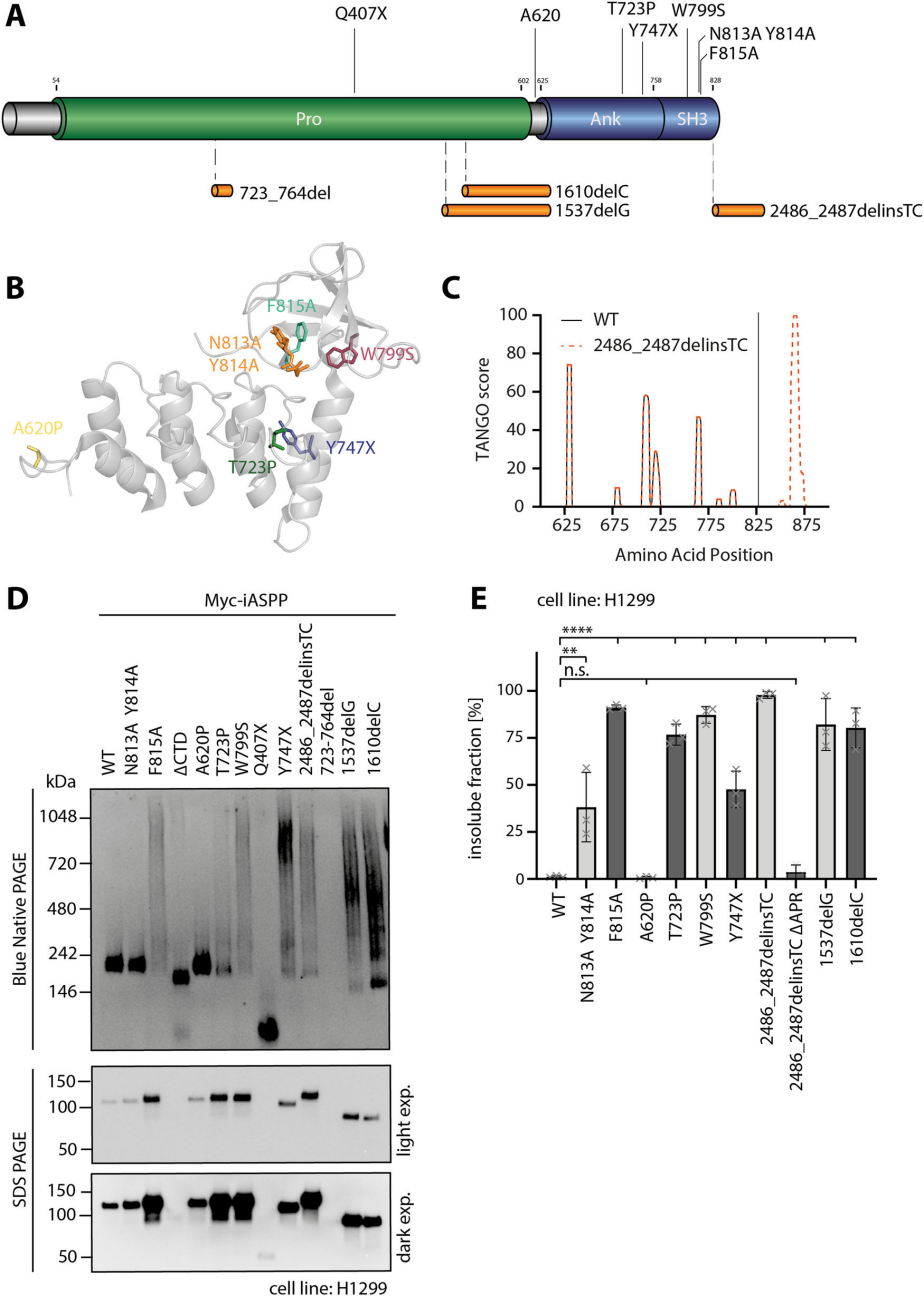
Cardiomyopathy related iASPP mutants are aggregation prone. **A** Schematic illustration of the structure of iASPP and localization of published cardiomyopathy associated mutations. Alternative C-termini resulting from frameshift mutations are shown in orange. **B** Crystal structure of the iASPP CTD (PDB: 2VGE) with highlighted missense mutations represented in stick mode. **C** Aggregation propensity prediction for the iASPP CTD using TANGO analysis. The frameshift mutation 2486_2487delinsTC leads to a 5’UTR extension predicted to be highly aggregation prone. **D** Blue Native PAGE (upper) and SDS PAGE (lower) of Myc-tagged iASPP WT and the indicated mutants. Proteins were expressed in transiently transfected H1299 cells and Western Blots were analyzed using an anti-Myc antibody. SDS PAGE was visualised using light and dark exposure (exp.). The F815A mutant is an artificial mutant used as a control mutant with a destabilized hydrophobic core. **E** Insoluble fractions of Myc-tagged iASPP WT or mutants. Proteins were expressed in transiently transfected H1299 cells and solubility was analysed via Western Blot. (Mean ± SD. *n* = 3, ordinary one-way ANOVA, n.s. *P* > 0.05; *P ≤ 0.05; **P ≤ 0.01; ***P ≤ 0.001; ****P ≤ 0.0001).

iASPP mutations linked to cardiomyopathy are typically homo- or compound heterozygous and appear across the entire protein (Fig. 1A, B). In principle three different classes of mutations can be identified (Supplementary Table 1). The first class contains mutations occurring in the unstructured N-terminus, such as frameshift mutations 723_764del, 1610delC and 1537delG as well as the nonsense mutation Q407X. All of them cause premature termination and consequently, a loss of the CTD.

The second class comprises a single missense mutation: A620P. This is the only heterozygous mutation and exchanges a residue in the short sequence directly adjacent to the CTD (Supplementary Figure 1A). This sequence contains a degenerate SH3 motif which was shown to mediate intermolecular interaction with the SH3 domain of another iASPP molecule [17]. Substitution of alanine with proline converts this sequence to a canonical SH3 class II motif, potentially increasing the affinity towards its own CTD and other SH3-domain containing proteins. We measured the affinity of a peptide spanning the sequence around the mutation to the iASPP CTD using Isothermal Titration Calorimetry (ITC). The mutant indeed binds almost six times stronger to the iASPP CTD than the WT peptide (K_D, A620P_ = 135 nM, K_D, WT_ = 743 nM). Both peptides were only weakly bound by the artificial N813A Y814A double mutant (K_D, N813A Y814A_ = ~ 5 µM) that is often used as a negative control for peptide binding to the SH3 domain of the CTD (Supplementary Fig. 1B).

The third class of mutations includes all missense and nonsense mutants of the CTD. While the lack of a complete CTD for mutations in the first class as well as the stronger auto-inhibition in the second class can explain the general observed loss of function, it was unclear how the diverse mutations within the CTD fit into this model and whether a single mechanism could potentially explain the development of this disorder. One hint towards such a mechanism was provided by the frameshift mutation 2486_2487delinsTC that skips the natural stop codon and elongates the native iASPP sequence by a 59 aa peptide. This peptide is highly hydrophobic and introduces an additional aggregation prone region (APR) to the CTD (Fig. 1C). We hypothesised that the other mutations in the CTD collectively induce aggregation as well. To test this, we overexpressed iASPP WT and its mutants in H1299 cells, and performed BN PAGE and SDS PAGE with the respective cell lysates (Fig. 1D, Supplementary Fig. 2). Samples were prepared without a centrifugation step after lysis to maintain all aggregates. We also included the frame shift mutations within the N-terminus to investigate their aggregation behaviour as well. iASPP WT, the artificial non-binding N813A Y814A mutant that lacks binding affinity to SH3 peptides as well as the A620P mutant showed no aggregation tendency, with comparable protein levels shown on the SDS polyacrylamide gel (Fig. 1D). The control mutant ΔCTD and the Q407X non-sense mutant, both missing the CTD and comprising only parts of the unfolded N-terminus displayed no aggregation behaviour either, yet they were invisible in SDS PAGE, indicating a very low protein level. The 723_764del mutant was neither visible in SDS PAGE nor in BN PAGE, potentially being directly degraded after translation. In contrast, the frameshift mutants 1537delG,1610delC and 2486_2487delinsTC as well as the nonsense mutant Y747X shifted to higher molecular weights in a smear-like manner, typical for aggregation. Interestingly, their protein levels were increased compared to iASPP WT. Similar results were obtained for the CTD missense mutants.

We further investigated whether cleaving the new C-termini of the frameshift mutations could rescue their aggregation behaviour. These truncated versions which contain only wildtype sequences showed wildtype-like behaviour on the BN gels, supporting the interpretation that the frameshifts are causing the aggregation (Supplementary Figure 1C). Notably, all proteins run at a molecular weight of approximately 250 kDa on the BN PAGE, twice as large as on the denaturing SDS‐PAGE. Although iASPP is proposed to form dimers via intermolecular interaction between the N- and the C-terminus, it is unclear if the observed shift in molecular weight is actually due to dimer formation, as all mutants including the ΔCTD control mutant that is missing the entire C-terminal domain run at a similar height. Therefore, it is more likely that the large unstructured N-terminus influences the migration behaviour in BN PAGE.

Next, we performed solubility studies with the mutants with detectable protein levels. iASPP WT and A620P were exclusively present in the soluble fraction, while all other mutants were predominantly found in the pellet fraction (Fig. 1E, Supplementary Fig. 1D). Even the artificial mutant N813A Y814A exhibited an increased insoluble fraction, despite appearing stable in BN PAGE. Cleaving the C-terminal elongation from mutant 2486_2487delinsTC resulted in its predominant presence in the soluble fraction, consistent with the suppression of aggregation seen in BN PAGE. Despite the observed differences in solubility and aggregation behaviour, the subcellular localization remained unaltered, with iASPP WT and the respective mutants being predominantly located in the cytoplasm (Supplementary Fig. 3).

### Reduced thermostability is the cause of the aggregation behaviour of mutated iASPP

**Fig. 2:**
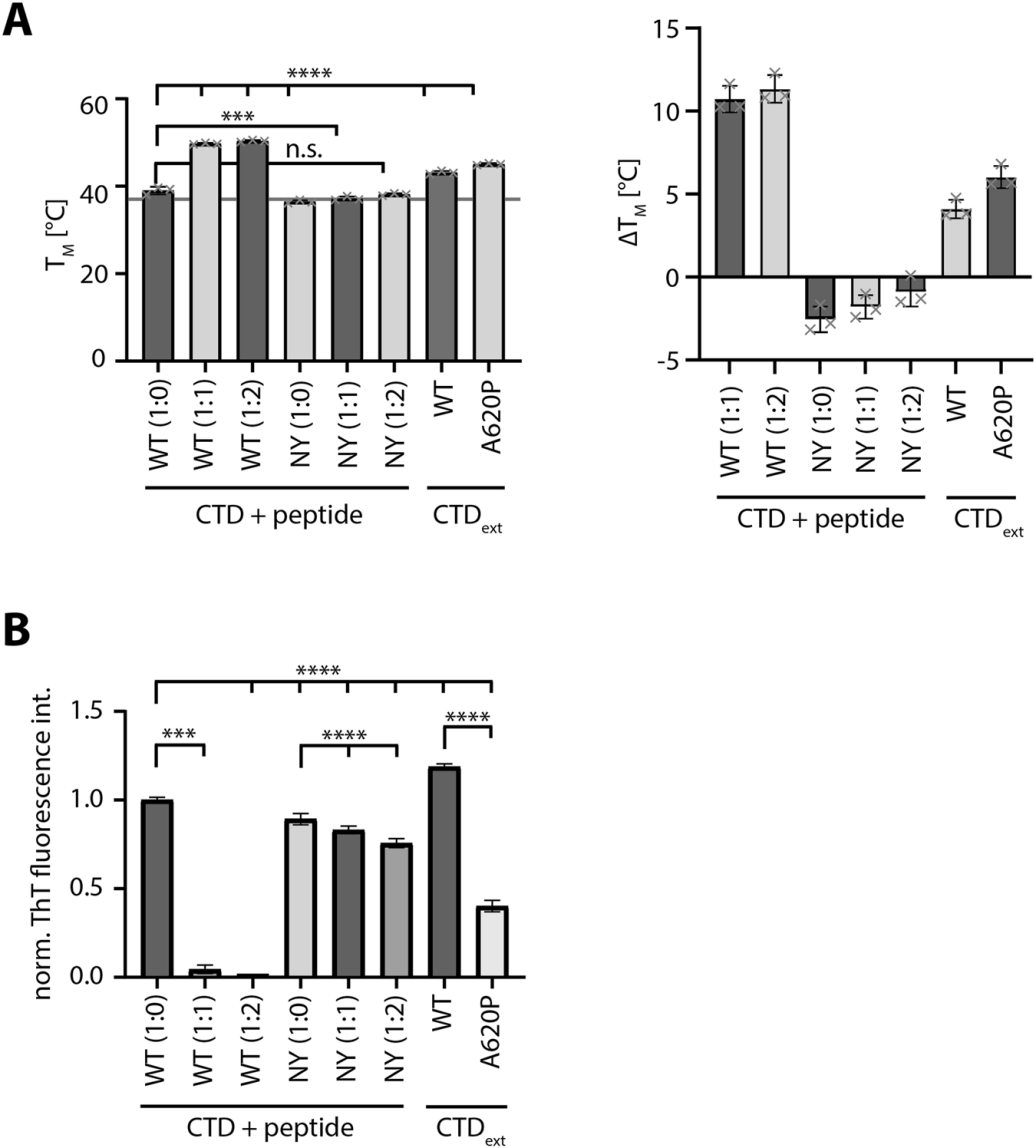
Thermostability of iASPP mutants is strongly reduced. **A** Melting temperatures of purified iASPP CTDs. Thermal shift assays were performed with iASPP WT and indicated mutants under increasing concentrations of PP1α peptide, if indicated, with absolute (left) and relative (right) values presented. The grey line marks 37 °C. CTD: AA625-828, CTD_ext_: AA608-828 (Mean ± SD. *n* = 3, ordinary one-way ANOVA, n.s. *P* > 0.05; *P ≤ 0.05; **P ≤ 0.01; ***P ≤ 0.001; ****P ≤ 0.0001). **B** Aggregation assay of purified iASPP CTDs using Thioflavin T (ThT). iASPP WT and mutants were incubated at 37 °C for 4 h. If indicated, increasing concentrations of PP1α peptide were added to the sample. Fluorescence signals, resulting from aggregate formation, were measured and the endpoint signal was determined. (Mean ± SD. *n* = 3, ordinary one-way ANOVA, n.s. *P* > 0.05; *P ≤ 0.05; **P ≤ 0.01; ***P ≤ 0.001; ****P ≤ 0.0001).

Aggregation and increased insolubility is most likely caused by a reduced thermostability of the mutants resulting in unfolding and aggregation through the exposure of hydrophobic sequences. To further examine this hypothesis, we attempted to measure the thermostability of the iASPP CTD including the missense mutations. However, expression of soluble protein in *E. coli* was only achieved for the non-aggregating iASPP WT, N813A Y814A and A620P while the other missense mutants were predominantly present in the pellet fraction, demonstrating their instability (Supplementary Fig. 4A). The successfully expressed and purified iASPP constructs were analysed by thermal shift assay to determine their melting temperature (Fig. 2A). Melting temperature of the iASPP CTD WT was only slightly above body temperature (T_M_ = 39.00 ± 0.9 °C), demonstrating its metastable state under physiological conditions. Addition of an interacting peptide raised the melting temperature by 10 °C, indicating that iASPP needs to form complexes with its binding partners in order to be stabilized. Consistently, the N813A Y814A control mutant had a melting temperature similar to iASPP CTD WT, yet the addition of a peptide failed to increase its stability above 38 °C. As the A620P mutation is adjacent to the first AR but not part of the CTD (starting from L625), we extended the CTD construct by 17 additional amino acids N-terminally to the CTD (referred to as CTD_ext_) which includes the auto-binding degenerate SH3 motif. Interestingly, addition of the auto-binding sequence alone was sufficient to increase the thermostability by 4 °C (T_M_ = 43.11 ± 0.4 °C) and was even higher for the A620P mutant (T_M_ = 45.03 ± 0.2 °C).

To assess the stability of the iASPP variants at physiological temperature over time, we performed an aggregation assay using the fluorescent dye Thioflavin T (Fig. 2B, Supplementary Figure 4B). Commonly used in Alzheimer’s research to visualize amyloid plaques, Thioflavin T is binding to aggregates resulting in an increased fluorescent signal. We incubated iASPP CTD WT and N813A Y814A with Thioflavin T at 37 °C and measured the fluorescent signal over the time course of 4 h. Addition of an interacting peptide prevented WT aggregation, while it had marginal effects on the N813 A Y814A control mutant. We then performed the same experiment for the CTD_ext_ of WT and A620P mutant. In line with its higher melting temperature, A620P formed significantly less aggregates than iASPP CTD WT. The aggregation potential of CTD_ext_ WT was slightly increased compared to CTD WT, despite having a higher melting temperature.

### Aggregating iASPP mutants induce a heat shock response and lose the ability to regulate NF-kB

**Fig. 3:**
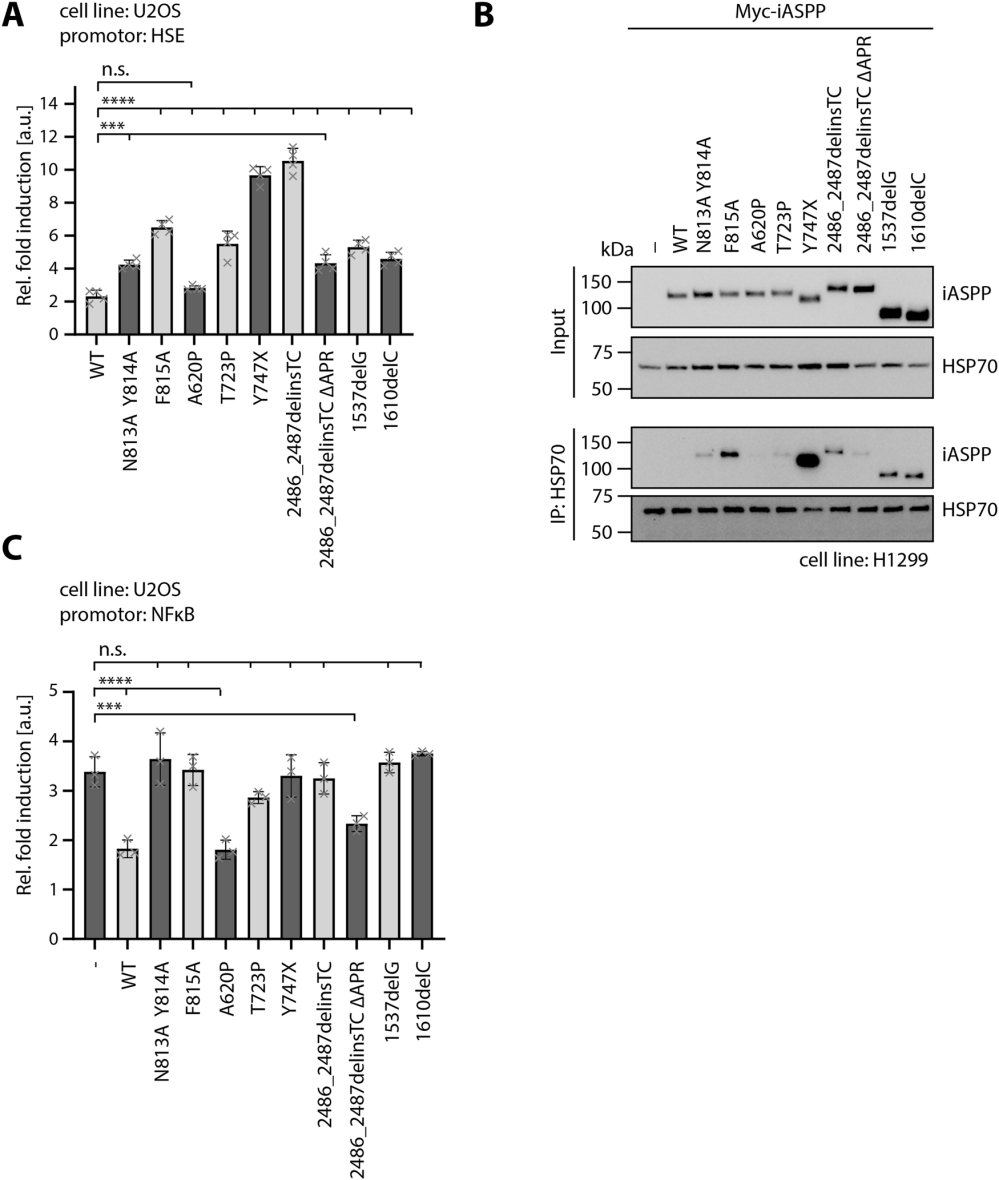
iASPP mutants trigger heat shock response and lose their ability to regulate NF-ĸB. **A** Luciferase reporter assay for detection of heat shock factor (HSF) activity using an artificial HSE promotor. U2OS cells were transiently transfected with the luciferase reporter plasmid, along with either an empty vector control, iASPP WT or a mutant. Measured activity was normalized to the empty vector control. (Mean ± SD. *n* = 3, ordinary one-way ANOVA, n.s. *P* > 0.05; *P ≤ 0.05; **P ≤ 0.01; ***P ≤ 0.001; ****P ≤ 0.0001). **B** Co-immunoprecipitation of endogenous HSP70 and Myc-tagged iASPP WT or mutants transiently transfected in H1299 cells. HSP70 was immunoprecipitated with an anti-HSP70 antibody and interaction with Myc-iASPP was detected using an anti-Myc antibody. *n* = 3. **C** Luciferase reporter assay for detection of p65 activity using an NF-ĸB promotor. U2OS cells were transiently transfected with the luciferase reporter plasmid, along with either an empty vector control, iASPP WT or a mutant. 24 h after transfection, cells were treated with 50 ng/ml TNFα for 6 h. Measured activity was normalized to the untreated empty vector control. (Mean ± SD. *n* = 3, ordinary one-way ANOVA, n.s. *P* > 0.05; *P ≤ 0.05; **P ≤ 0.01; ***P ≤ 0.001; ****P ≤ 0.0001).

Accumulating protein aggregates can induce a heat shock response (HSR) that upregulates the expression of heat shock proteins crucial for protein stabilization, transport and degradation. As most of the iASPP mutants tend to aggregate, we measured the resulting heat shock response in transiently transfected U2OS cells with the frameshift and selected missense mutants (Fig. 3A, Supplementary Fig. 5A). Upon HSR activation, the transcription factor heat shock factor 1 binds to the artificial heat shock element (HSE) in the promotor of the firefly luciferase promotor plasmid. As expected, all mutants expect for A620P elicit higher HSR signals than iASPP WT. The increase was most pronounced for 2486_2487delinsTC and Y747X, despite the latter forming the least insoluble aggregates in the solubility assay. Cleaving the C-terminal extension of 2486_2487delinsTC reduced the HSR level but was not able to fully restore it to the level observed for WT.

Given that upregulation of the HSR implies the involvement of chaperones, we performed co-immunoprecipitation experiments with transiently transfected H1299 cells and endogenous HSP70 (Fig. 3B, Supplementary Figure 2). To eliminate any insoluble aggregates, all cell lysates were centrifuged prior to immunoprecipitation. As anticipated, all iASPP mutants except for A620P interacted with HSP70, albeit to varying extents. Notably, the signal detected for Y747X was the strongest, consistent with Y747X showing aggregation in the BN PAGE but having the highest amount of soluble protein in the solubility assay.

We further tested the ability of iASPP mutants to inhibit p65 transcriptional activity in TNFα treated U2OS cells (Fig. 3C, Supplementary Fig. 5B). Compared to a non-treated control, p65 activity increased upon TNFα activation. As expected, transiently transfection with iASPP WT and rescue mutant 2486_2487delinsTC ΔAPR inhibited its binding to the NF-ĸB promotor. Notably, A620P was also able to decrease p65 activity, suggesting that increased auto-inhibition does not affect their interaction. No significant effects were observed for any of the other disease-related mutants indicating that these mutants are unable to interact with p65.

### iASPP mutations in human cancer do not commonly lead to aggregation

**Fig. 4:**
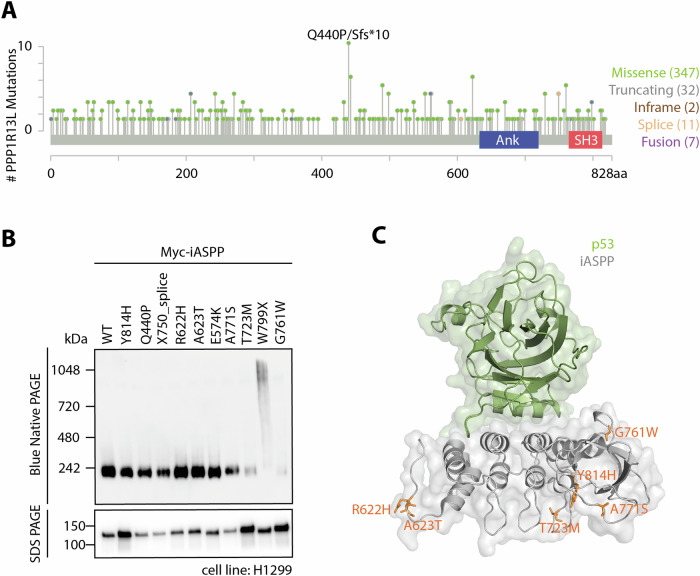
iASPP cancer mutants do not induce aggregation. **A** Lollipop diagram showing localization and frequency of reported iASPP mutations in cancer (taken from cBioPortal: https://bit.ly/3yrAWvA). **B** BN PAGE (top) and SDS PAGE (bottom) of selected iASPP mutants transiently transfected into H1299 cells. Western Blots were evaluated using an anti-Myc antibody. **C** Superimposed crystal structures of the iASPP CTD (PDB: 2VGE; AA616-823) and the iASPP CTD bound to the p53 DBD (PDB: 6RZ3; iASPP: AA657-823; p53: AA91-291). Mutations included in (**B**) are visualised as sticks in orange.

In addition to p65, iASPP is known to bind to p53 and was originally described as a p53 inhibitor. Since tumour formation often results from malfunctions in the regulation of p53, we wondered whether iASPP might also be mutated in cancer and if these mutations lead to protein aggregation. To address this question, we searched for genomic alterations in a human carcinoma database comprising 217 independent studies with over 70,000 samples (https://bit.ly/3yrAWvA). We identified 399 mutations in 354 patients, which however did not accumulate in any hotspot (Fig. 4A). The only mutation standing out was Q440P being reported in 8 different patient samples and mainly occurring in melanoma. We selected several mutations from different cancer types for BN PAGE analysis, including mutations found in cutaneous melanoma (X750_splice, R622H), colorectal adenocarcinoma (A623T), desmoplastic melanoma (E574K), hepatocellular carcinoma (A771S), prostate adenocarcinoma (T723M) as well as lung adenocarcinoma (W799X and G761W) (Supplementary Table 2). Interestingly, none of the analysed mutants except for W799X resulted in aggregation (Fig. 4B, Supplementary Fig. 2) and neither of them are localized within the interaction interface between iASPP and p53 (Fig. 4C). It is therefore unclear if these missense mutations occur randomly or if the underlying mechanism is different from the cardiomyopathy inducing germline mutants.

## Discussion

In this study, we classified the currently published iASPP germline mutations associated with cardiocutaneous syndrome and examined their impact on thermostability and aggregation behaviour. While all N-terminal mutants result in premature termination and consequently, a loss of the CTD, we demonstrate that mutations within the CTD share a common disease mechanism that is based on aggregation. Since the melting temperature of iASPP WT CTD is just above normal body temperature, mutations within the hydrophobic core of the CTD further decrease its thermostability, leading to higher fractions of unfolded protein and exposure of aggregation prone sequences. Interestingly, analysis of patient derived fibroblasts has suggested that cells with aggregating iASPP frameshift or nonsense mutations manage to decrease the iASPP concentration, thus potentially reducing deleterious “gain of function” effects of the aggregates [12, 15].

Since all mutations cause loss of functionality, the interaction with endogenous binding partners is likely to be impaired. As a proof-of-concept, we performed a transactivation assay with p65, a member of the NF-ĸB family. iASPP mutants were not able to inhibit p65 activity upon TNFα activation in transiently transfected U2OS cells. Consistent with our finding, skin-derived fibroblasts from iASPP mutant patients were shown to have an NF-ĸB-dependent increase in mRNA levels of inflammatory cytokines upon LPS stimulation [12]. Elevated cytokine levels were also observed in LPS-treated murine iASPP KO cardiomyocytes and the hearts of wa3 mice [12]. Notably, overactivity of p65 is a known cause for the development of cardiomyopathy [18].

iASPP also plays a crucial role in the maintenance of desmosome integrity, keeping desmoplakin in a dephosphorylated and insoluble state. This function is potentially mediated via PP1, known to form trimeric complexes with iASPP and its target proteins by binding to its SH3 domain (as seen for p53). In a mouse model, cell specific KO of iASPP in keratinocytes and cardiomyocytes leads to reduced epithelial cell adhesion and cardiac dysfunctions, respectively [10]. Additionally, germline iASPP KO in mice impairs cellular integrity in the myocardium and causes cardiomyopathy, reminiscent of the phenotype observed for desmoplakin-deficient mice [9].

Cutaneous symptoms could also result from impaired regulation of p63, a p53 family member that is highly expressed in the basal compartment of epithelial tissues [19]. iASPP co-localizes with p63 in the nucleus of human basal epithelial cells and is involved in the regulation of p63 and AP-1 target genes important for the maintenance of tissue homeostasis [8]. Since KO of p63 in epithelial cells decreases the transcription of adhesion-related genes [20], co-aggregation with the iASPP mutants might induce similar effects. Interestingly, mutations in the SAM domain of p63 have been shown to cause the Ankyloblepharon-ectodermal Defects-cleft Lip-palate (AEC) Syndrome [21] which is characterized by skin fragility. The underlying mechanism is based on unfolding of the SAM domain followed by the exposure of aggregation-prone sequences usually hidden in the three-dimensional structure and finally aggregation [22].

The only mutant differing in its molecular mechanism is the missense mutation A620P, causing increased stability and stronger binding to iASPP WT. Notably, this mutation is the only heterozygous one. Since the respective patient presents with the same phenotype as the homozygous mutants, A620P might have a dominant-negative effect on iASPP WT due to enhanced auto-inhibition. Although we did not see any effect on the activity of p65, A620P could potentially compete with other endogenous ligands such as p63 and PP1, effectively resulting in a loss-of function. The canonical SH3 motif completed by the alanine to proline exchange might likewise be bound stronger by any other SH3 domain containing protein in the cell, however, the relevant binding partner would remain elusive since enhanced interaction with the currently known binding partners would not explain the observed phenotype.

Given that iASPP acts as a negative regulator of p53, a key driver of cancer, we sought to examine the aggregation behaviour of iASPP mutations in different cancers. The iASPP CTD is known to bind the p53 DBD, displacing the p53 L1 loop without disturbing the remaining sequence specific DNA contacts [23]. As a result, this interaction modulates the transcriptional program of p53, typically assigning iASPP the role of a tumour enhancer. However, we could recently show that iASPP can suppress inflammation-driven skin tumorigenesis in mice independently of p53, via a JNK-iASPP-AP-1 axis [8]. Analysis of iASPP mutations in a wide range of cancers did not reveal particular hotspot mutations and, unlike the mutations associated with cardiomyopathy, most of the mutants selected for further characterisation neither aggregated nor reside at the hydrophobic core or binding interface between iASPP and p53. Whether these mutations are otherwise involved in tumorigenesis or arise spontaneously without any implications for tumor development remains to be further investigated.

## Materials and Methods

### Cloning and mutagenesis

PCR generated inserts of ASPPs were subcloned into pcDNA3.1(+)-Myc vector for transient transfection of human cells or a pET-15b-His10-TEV (N-terminal His10-tag followed by TEV protease cleavage site) vector for recombinant *E. coli* expression using BamHI and XhoI restriction sites. Plasmids used for luciferase reporter assays were based the pRL-CMV and the pGL3-Basic vector. Promotors in the pGL3-Basic vector were adapted to the respective experiment (NF-ĸB: 5x GGGGACTTTCC; HSE: 3x AGAACGTTCTAGAAC) and subcloned using NheI and XhoI restriction sites. iASPP mutants were obtained by site-directed mutagenesis on pcDNA3.1(+)-Myc-iASPP. For the solubility assay, the CMV promotor was exchanged to an artificial minimal promoter 10RPU with lower expression [24] between the MfeI and NheI restriction sites. All gene inserts are devoid of the initial methionine to avoid alterative translation initiation.

### Cell culture

H1299 cells were cultured in RPMI 1640 medium supplemented with 10% (v/v) foetal bovine serum (FBS), 100 U/ml penicillin and 10 µg/ml streptomycin. For cultivation of U2OS cells, DMEM medium was supplemented with 1% pyruvate, 10% (v/v) foetal bovine serum (FBS), 100 U/ml penicillin and 10 µg/ml streptomycin. For maintenance, cells were incubated at 37 °C under an 5% CO_2_ atmosphere and regularly tested for mycoplasma (LookOut Mycoplasma PCR detection kit, Sigma-Aldrich).

For transfection, cells were treated with Lipofectamine 2000 (Thermo Fisher Scientific) according to the manufacturer’s protocol. In brief, cells were seeded per into well plates (0.8*10^5^ cells for 12 well plate, 2.0*10^5^ cells for 6 well plate, Greiner BioOne) using antibiotics free media. 24 h after seeding, cells were transfected with Lipofectamine 2000 (2 µl for 12 well, 5 µl for 6 well) and a fixed amount of DNA (800 ng for 12 well, 2000 ng for 6 well), equally distributed among plasmids.

### SDS PAGE and Western blotting

Samples for SDS Page were collected in 2x SB buffer (Laemmli buffer with 5% β-mercaptoethanol) and boiled at 95 °C for 5 min before loading them onto a 4–15% Mini-PROTEAN TGX gel (Bio-rad).

For western blotting, proteins were transferred into a PVDF membrane using the TransBlot Turbo transfer system (Bio-rad) according to manufacturers’ instructions. Blots were blocked with 5% (w/v) skim milk in TBSt (TBS buffer with 0.1% Tween-20) for 1 h at RT before incubating them with the respective primary antibody overnight at 4 °C. The following day, blots were washed 3 × 10 min with TBSt and incubated with HRP-coupled secondary antibody for 1 h at RT. After repeated washing with TBSt, blots were visualised using Amersham ECL Prime western blotting detection reagent and the ChemiDoc imaging system (Bio-Rad). Signals were quantified using the Image Lab 6.1 software from Bio-Rad. All antibodies are listed in Supplementary Table 3.

### Blue Native PAGE

24 h after transfection, cells were resuspended in lysis buffer (20 mM Tris-HCl (pH 7.5), 150 mM NaCl, 2 mM MgCl_2_, 20 mM CHAPS, 1 mM DTT, protease inhibitor, PhosStop) supplemented with benzonase. After incubation for 1 h on ice, samples were mixed with 3x BN-PAGE (60% glycerol, 15 mM Coomassie Brilliant Blue G-250) and loaded onto a 3–12% Novex Bis-Tris gradient gel (ThermoFisher Scientific). Sample separation was performed for 1 h at 150 V, followed by 1.5 h at 250 V using the NativePAGE Bis-Tris Gel System at 4 °C. Subsequent blotting using the XCell II blot system (Invitrogen) was followed by destaining the PVDF membrane with methanol and fixing it with 8% acetic acid. Blocking and incubation with the respective antibodies was performed as described for western blotting.

### Solubility assay

24 h after transfection of the indicated iASPP mutants, H1299 cells were lysed in 100 µl Triton buffer (buffer basis: 20 mM Tris (pH 7.5), 150 mM NaCl, 1 mM DTT, 2 mM MgCl_2_; Triton buffer: buffer basis, protease inhibitor, 1% Triton X-100) supplemented with benzonase and incubated for 1 h on ice. Lysate was cleared by centrifugation at 13,000 rpm and 4 °C. The supernatant was removed and mixed with 5x SB. The remaining pellet was resuspended in 100 µl SDS buffer (buffer basis, 1% SDS) and incubated for 15 min at room temperature before cells were centrifuged and the supernatant was mixed with 5x SB again.

### Immunoprecipitation

24 h after transfection, cells were lysed in RIPA buffer (50 mM HEPES (pH 7.5), 150 mM NaCl, 1% NP-40, 1% sodium deoxycholate, 1 mM DTT; 1 mM MgCl_2_) supplemented with protease inhibitor, phosphatase inhibitor and benzonase. Following incubation for 30 min on ice, cell suspension was centrifuged at 13,000 x g for 15 min. Input samples were taken and the remaining supernatant was diluted 1:4 with IP buffer (50 mM HEPES pH7.5, 200 mM NaCl, 0.1% Tween-20), adding 0.5 µg of the respective antibody. After incubation overnight at 4 °C, 10 µl magnetic Protein G Dynabeads (Thermo Fisher Scientific) were added and samples were incubated again for 3 h at 4 °C. Samples were washed 4x with IP buffer and beads were resuspended in LDS buffer supplemented with 0.5 M DTT and incubated at 70 °C for 10 min before the supernatant was transferred into a new tube.

### Luciferase reporter assay

H1299 cells were transfected with a pRL-CMV plasmid and a pGL3 Basic plasmid under a HSE or NF-ĸB promotor and the respective iASPP mutant or an empty vector control.

TNFα Luciferase reporter assay was performed 24 h after transfection, using the Dual-Glo luciferase assay system (Promega). For p65 studies, cells were additionally treated with 50 ng/ml TNFα for 6 h, before following the manufacturer’s protocol. Briefly, cells were harvested and resuspended in fresh medium before taking input samples and transferring the remaining cells into a Nunclon^TM^ Delta Surface 96-well plate (Thermo Fisher Scientific). The cell suspension was incubated with Firefly substrate for 10 min at RT before the luminescence signal was measured using a Spark multimode microplate reader (Tecan). Subsequently, Renilla substrate was added and the procedure was repeated.

Measurements were performed three times in technical quadruplicates. The ratio of the Firefly to Renilla luciferase signal was calculated for each technical replicate before determining the mean and normalising it to the empty vector control.

### Immunofluorescence (IF) staining

For IF staining, U2OS cells were seeded on coverslips and transfected with Myc-tagged iASPP WT and respective mutants. 24 h after transfection, cells were washed several times with PBS and fixed in 4% paraformaldehyde for 10 min at RT. After another washing step, cells were permeabilised with permeabilisation buffer (PBS supplemented with 0.1% Triton X-100) for 15 min at RT, before being blocked with blocking buffer (PBSt supplemented with 1% BSA) for 30 min at RT. Coverslips were then incubated with primary antibody overnight at 4 °C in a humidified chamber. After washing them three times with PBSt (PBS supplemented with 0.1% Tween-20) the next day and incubating them for 1 h with the appropriate secondary antibody at RT, cells were washed again with PBSt before being mounted onto microscope slides using Mowiol (Carl Roth) supplemented with DAPI (Thermo Fisher Scientific). Cells were imaged using the confocal microscope Leica TCS SP5.

### *E. Coli* expression

*E. Coli* BL21(DE3) Rosetta cells were transformed with the respective expression plasmid and grown in 2xYT medium supplemented with 100 µM zinc acetate at 37 °C until an optical density (OD) of 0.8 was reached. After induction with 0.5 mM IPTG, cells were incubated at 20 °C overnight. For harvesting, cells were centrifuged at 6700 x g and the pellet was resuspended in IMAC A buffer (25 mM Tris pH 7.8, 200 mM NaCl, 20 mM β-mercaptoethanol, 5% glycerol, 25 mM Imidazole) supplemented with a DNase/RNase mix, lysozyme and self-made protease inhibitor. Following incubation for 1 h on ice and subsequent sonification, lysate was centrifuged at 27,000 x g and the supernatant underwent further purification.

For solubility analysis, *E. coli* cells were grown and harvested as described above. After lysis and subsequent centrifugation, samples were taken from the pellet and the supernatant, respectively, mixed with 2x SB buffer and subjected to SDS PAGE.

### Protein purification

All purification steps were performed at 4 °C using an ÄKTA Purifier chromatography system (Cytiva). After expression as described above, His-tagged proteins were loaded onto a HiTrap IMAC FF column (Cytiva) equilibrated with IMAC A buffer before being eluted with IMAC B buffer (IMAC A buffer with 400 mM Imidazole). Protein elutions were supplemented with His-tagged TEV protease to cleave off the tag and were simultaneously dialysed against IMAC A buffer overnight at 4 °C. The following day, protease and cleaved tag were removed by performing reverse Ni-affinity purification. In preparation of IEX, salt concentration of the protein solutions was reduced to 100 nM by diluting them with IEX buffer A (25 mM Tris pH7.8, 50 mM NaCl, 20 mM ß-ME, 5% Glycerol) before loading them onto a HiTrap Q column (Cytiva). Proteins were eluted by applying a salt gradient mixing IEX A buffer and IEX B buffer (25 mM Tris (pH 7.8), 1000 mM NaCl, 20 mM ß-ME, 5% Glycerol) over 20 column volumes. For the final purification step, proteins were subject to size exclusion chromatography using a Superdex 75 SEC column (Cytiva). All proteins were stored in assay buffer (25 mM HEPES (pH 7.5), 150 mM NaCl, 0.5 mM TCEP), snap-frozen in liquid nitrogen and stored at −80 °C until further processing.

### Aggregation Assay

Aggregation studies were performed with Thioflavin T, generating a strong fluorescence signal upon binding to aggregates. For sample preparation, 10 µM purified iASPP CTDs in assay buffer [50 mM HEPES (pH 7.5), 150 mM NaCl, 0.5 mM TCEP] were mixed with 25 µM ThT in black, non-binding 96 well plates (Greiner). Samples were incubated at 37 °C for 4 h while measuring the fluorescence signal in 1 min intervals at 485 nm.

### Thermal Shift Assay

Thermal shift assay (TSA) was performed to determine melting temperatures of purified iASPP CTDs. Samples were prepared by thawing and centrifuging the CTDs at 13,000 x g for 15 min at 4 °C before mixing the CTDs (20 µM in assay buffer) with SYPRO Orange (Thermo Fisher Scientific, 1:500 diluted in assay buffer) and PP1α (AA311-330) peptide (20 µM or 40 µM in assay buffer) if indicated. Measurements were performed in semi-skirted 96‐well PCR plates (Starlab) using a QuantStudio^TM^ 5 cycler (Bio-rad), starting at 4 °C and gradually increasing the temperature to 99 °C with a rate of 0.1 °C/s. Fluorescence signal was measured and melting temperature was determined by calculating the negative first deviation (-dF/dT) with GraphPad Prism 8.

### Isothermal titration calorimetry

Isothermal titration calorimetry (ITC) experiments were performed using a VP-ITC (MicroCal). Prior to measuring, dissolved iASPP CT peptides and purified ASPP CTDs were dialysed in ITC buffer (25 mM HEPES (pH 7.5), 150 mM NaCl, 500 μM TCEP) overnight at 4 °C. All measurements were set up at 12 °C and performed with 25 titration steps à 10 µl. 450 µM iASPP CT peptides were titrated to 30 µM purified ASPP CTDs. The resulting binding curve (corrected by the dilution heat of the respective ligand) was analysed with NITPIC and SEDPHAT, calculating the dissociation constant K_D_, the enthalpy (ΔH) and the entropy (ΔS) as well as the 95% confidence interval. Figures were prepared using GUSSI. Peptide sequences and all determined thermodynamic parameters are listed in Supplementary Table 4.

### Statistical analysis

If not indicated otherwise, experiments were repeated at least three times to determine mean and standard deviation. Comparison of independent data sets was performed using one-way analysis of variance (ANOVA) followed by Tukey’s HSD multiple comparison post hoc tests. All statistical calculations were done with GraphPad Prism 8.

## Supplementary information

SUPPLEMENTAL MATERIAL

Supplemental Figure 2

## Data Availability

Original data are available upon request.
