# Supplementary material for: Cardiocutaneous syndrome is caused by aggregation of iASPP mutants
Source: Cell Death Discov. 2024 Dec 18;10:497. doi: 10.1038/s41420-024-02265-z (PMC11655644; doi:10.1038/s41420-024-02265-z)
Supplement: Supplementary file 1 — SUPPLEMENTAL MATERIAL [file 41420_2024_2265_MOESM1_ESM.docx]

**­­Supplementary Figures and Figure legends**


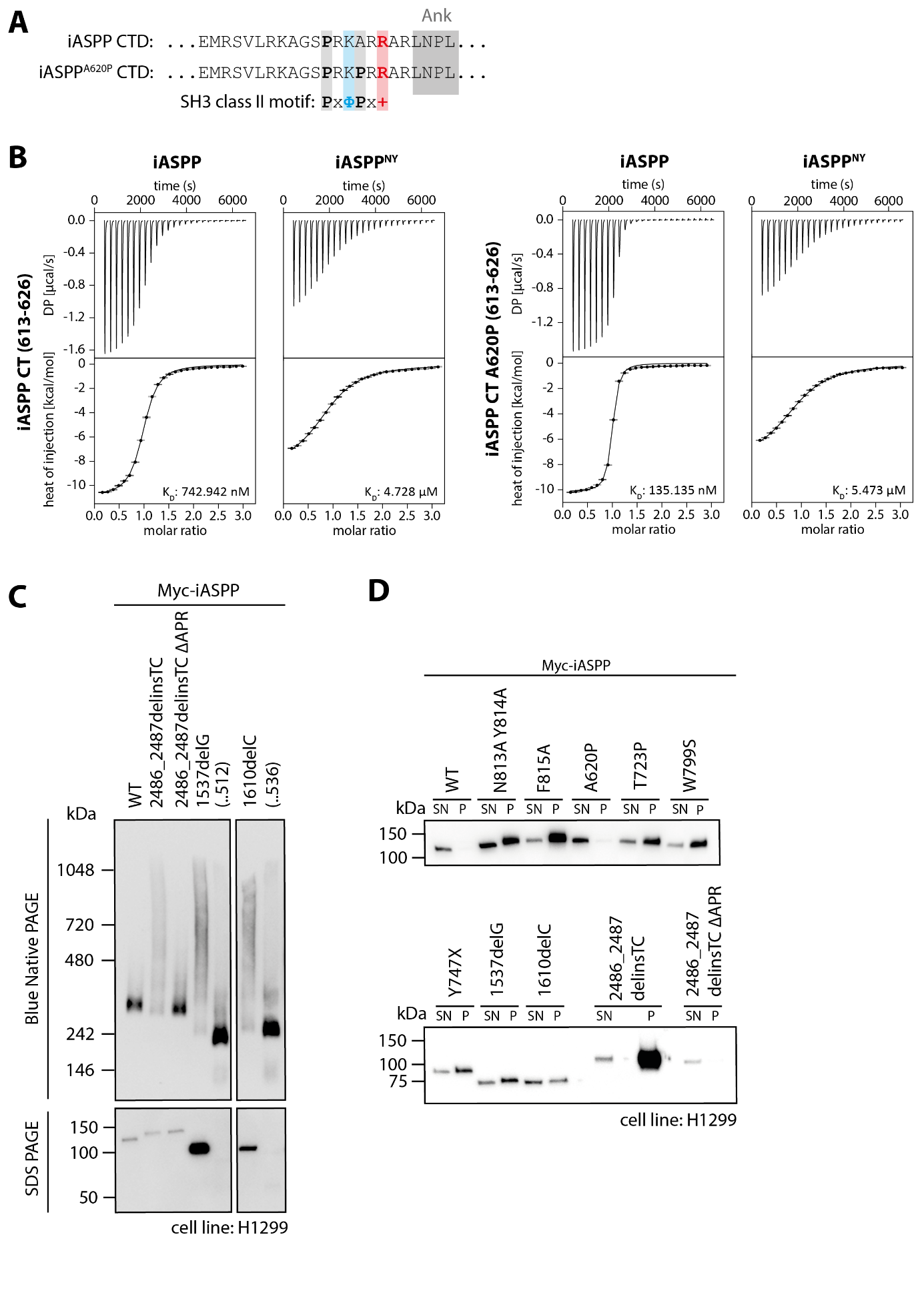


**Suppl. Fig. 1**

**A** Sequence comparison between iASPP WT and the A620P mutant. The point mutation introduces a canonical SH3 class II peptide adjacent to the first Ankyrin repeat (grey box). **B** ITC measurements of iASPP C-terminal (CT) peptide (AA613-626, WT or with the A620P mutation) titrated to purified iASPP CTD and the iASPP N813A Y814A double mutant. Thermograms are shown in the top panel while integrated isotherms are displayed in the bottom panel. Data were analyzed assuming a single‐site binding model and the resulting fit is shown as a solid black line with the respective K_D_ indicated. Additional thermodynamic parameters are provided in Table S3. **C** Native PAGE (upper panel) and SDS PAGE (lower panel) of Myc-tagged iASPP WT, the indicated mutants and their respective rescue mutants. Proteins were expressed in transiently transfected H1299 cells and WB were analysed using anti-Myc antibody. **D** Western Blot of solubility assay with Myc-tagged iASPP WT or mutants shown in Fig. 1E. Proteins were expressed in transiently transfected H1299 cells and soluble/insoluble fractions were determined via WB using anti-Myc antibody.


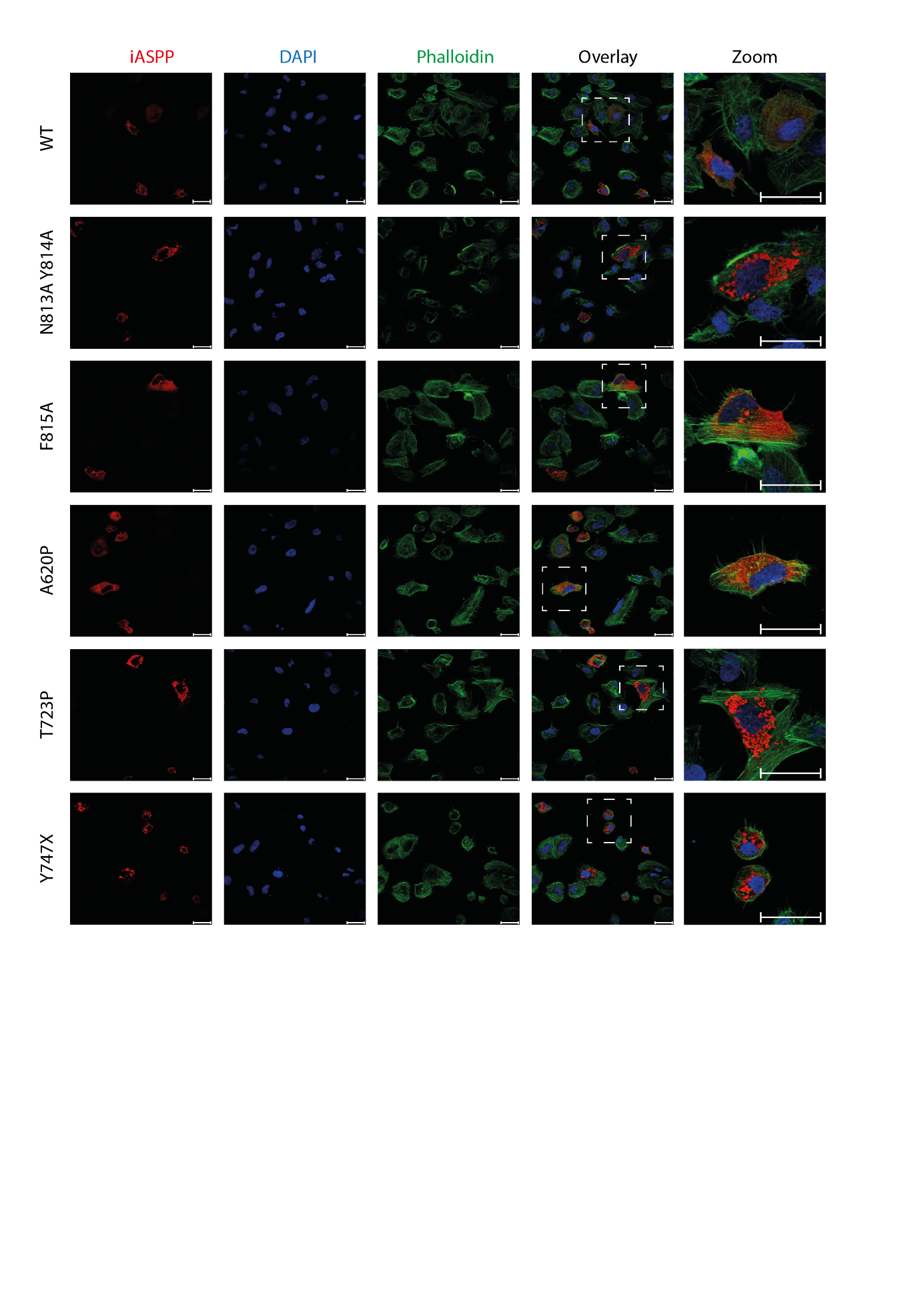


**Suppl. Fig. 3**

Immunofluorescence staining of U2OS cells transiently transfected with myc-tagged iASPP WT or mutants. Cells were co‐stained for nuclei (blue), actin filaments (green) and iASPP (red) with DAPI, fluorophore‐labelled phalloidin and anti‐Myc antibody. Images are shown individually and as overlays. White dashed boxes mark the magnified area. Scale bar: 30 μm.


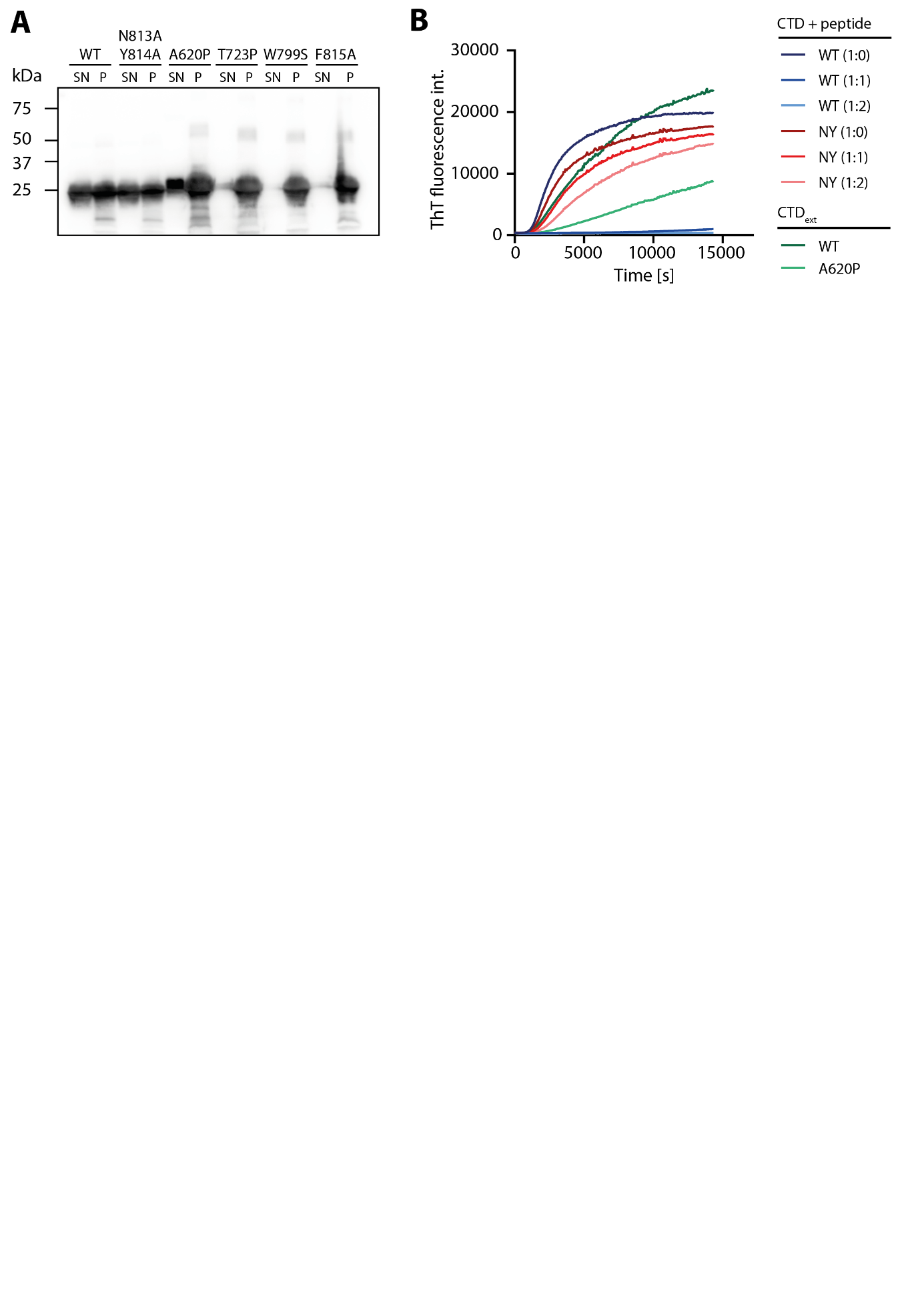


**Suppl. Fig. 4**

**A** Soluble and insoluble fractions of iASPP WT and indicated mutants expressed in *E. coli*. **B** Raw data of Thioflavin T assay. Purified iASPP CTDs were incubated at 37°C for 4 hours. If indicated, PP1α peptide was added in varying ratios. Fluorescence signals, resulting from aggregate formation, were measured.


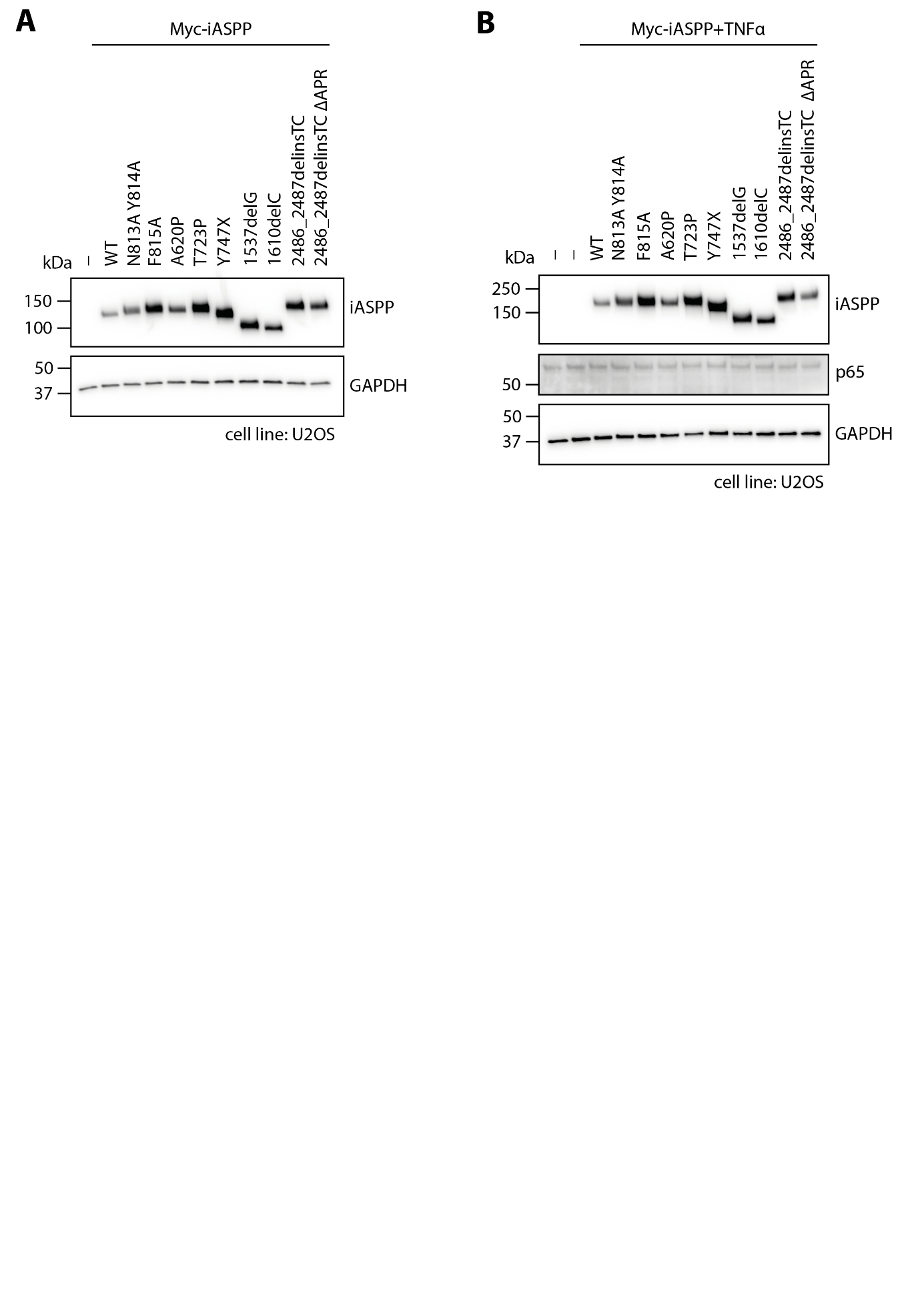


**Suppl. Fig. 5**

**A** Input levels of iASPP WT and indicated mutants used for the luciferase reporter assay shown in Fig. 3A. Western Blot signals were detected using an anti-Myc antibody. GAPDH was used as a loading control. **B** Input levels of iASPP WT and indicated mutants used for the luciferase reporter assay shown in Fig. 3C. p65 activity was triggered with TNFα, if indicated. Western Blot signals were detected using an anti-Myc antibody. Endogenous p65 levels were detected using an anti-p65 antibody. GAPDH was used as a loading control.

**Suppl. Table 1**

Summary of cardiomyopathy associated iASPP mutations. Mutations not used in this study are marked with a red box.

**Suppl. Table 2**

Number of reported cases and associated cancer types of selected iASPP mutations

(taken from cBioPortal: https://bit.ly/3yrAWvA).

**Suppl. Table 3**

Antibodies used in this study for Western blot (WB) and immunofluorescence staining (IF)

**Suppl. Table 4**

Peptide sequences used for ITC (upper panel) and determined thermodynamic parameters including 95% confidence intervals in brackets (lower panel).
