# Supplementary material for: Cardiocutaneous syndrome is caused by aggregation of iASPP mutants
Source: Cell Death Discov. 2024 Dec 18;10:497. doi: 10.1038/s41420-024-02265-z (PMC11655644; doi:10.1038/s41420-024-02265-z)

uncropped western blots for Fig. 1D - Replicate 1 (shown in figure)

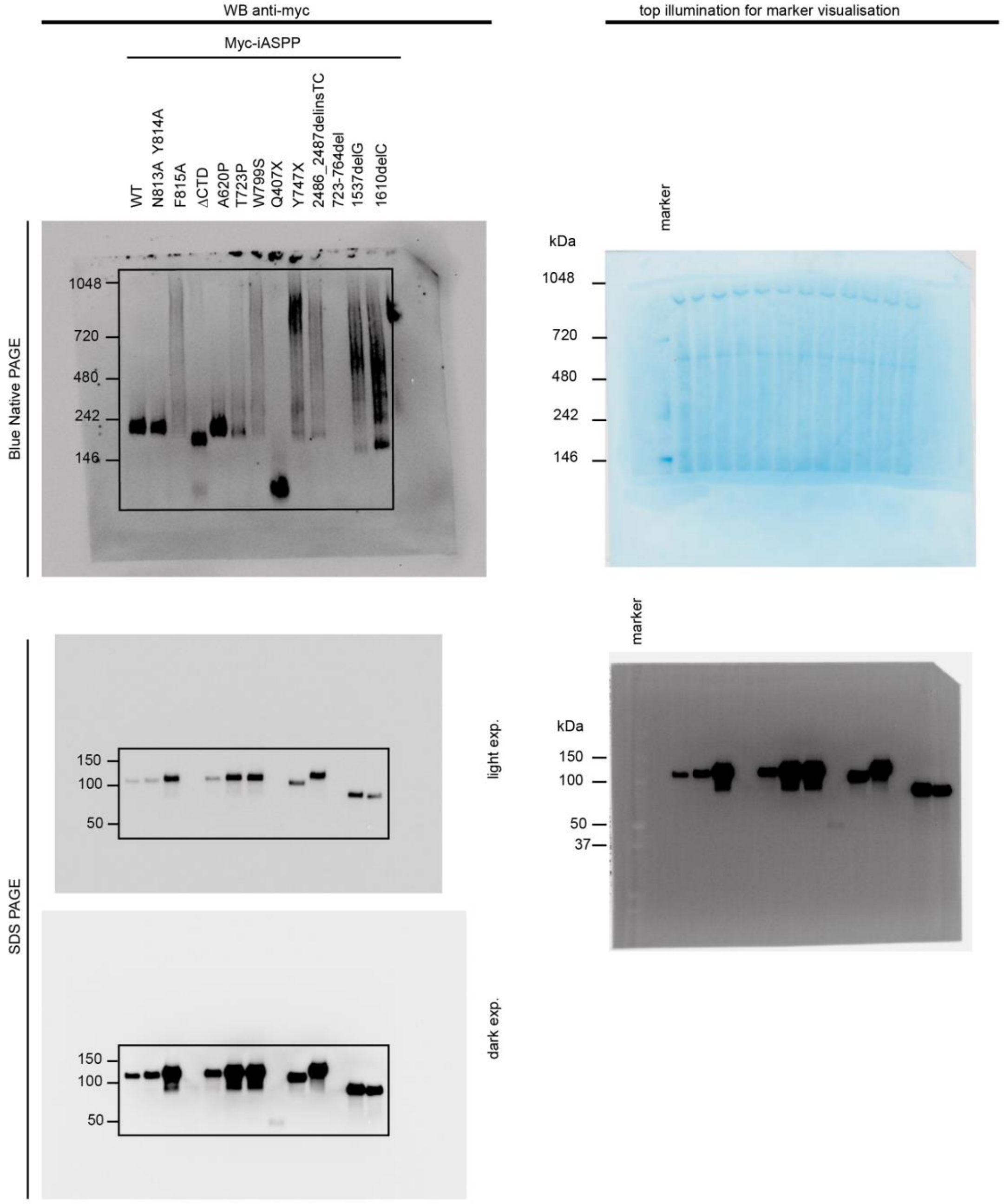

uncropped western blots for Fig. 1D - Replicate 2

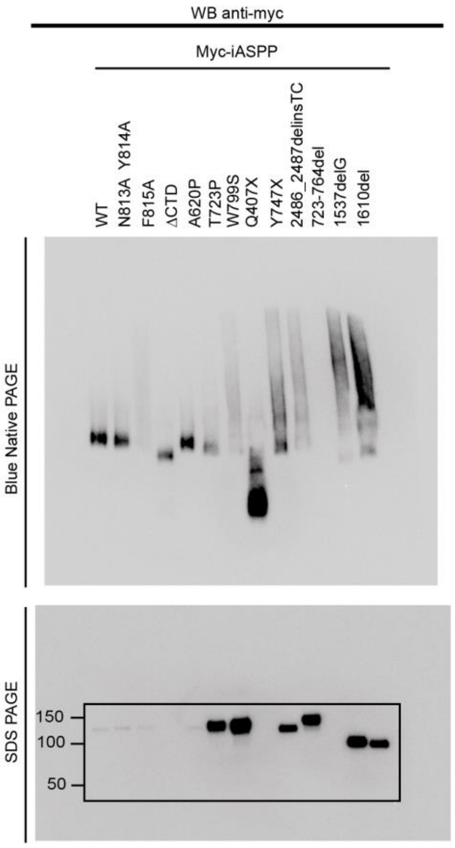

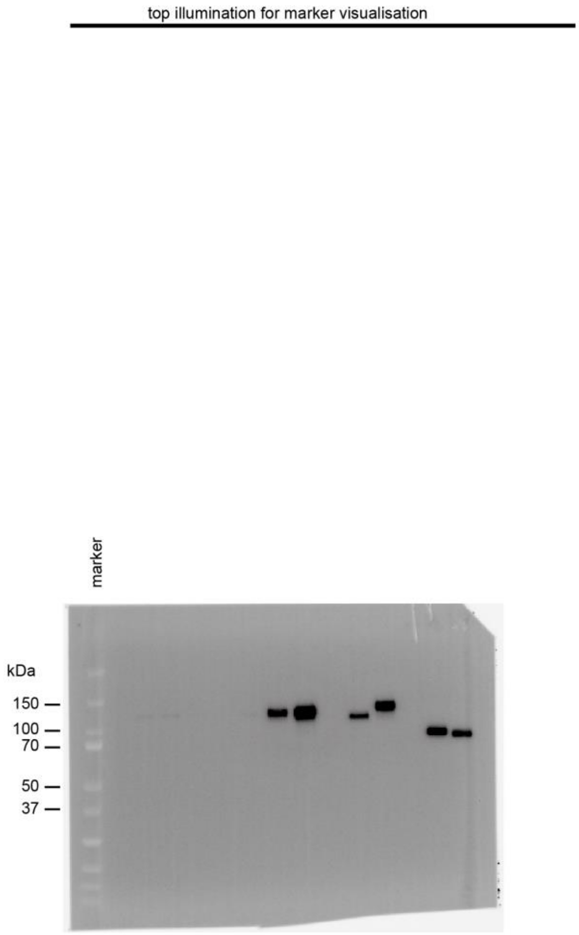

uncropped western blots for Supplementary Fig. 1C - Replicate 1 (shown in Figure)

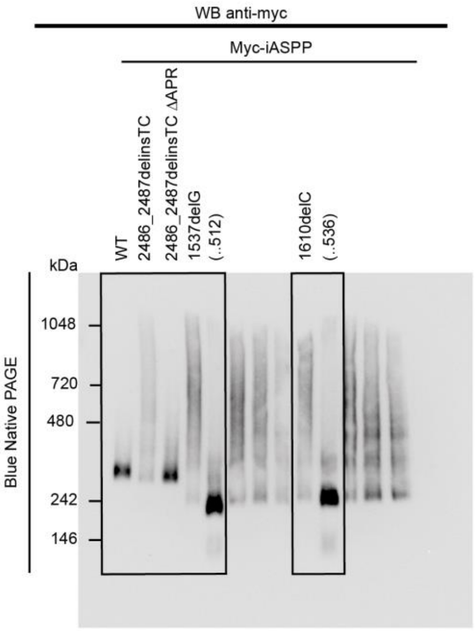

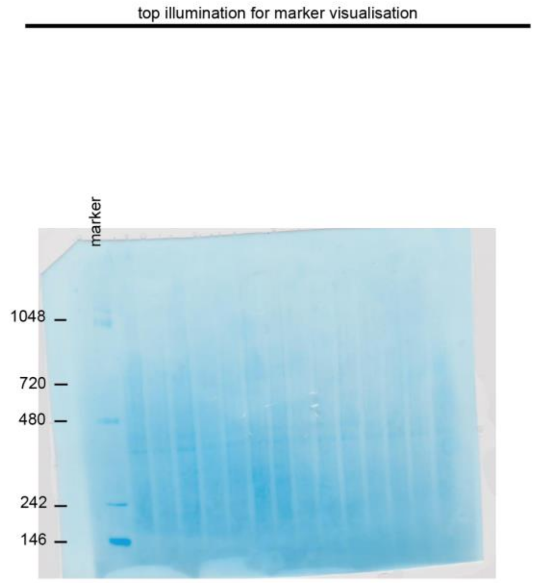

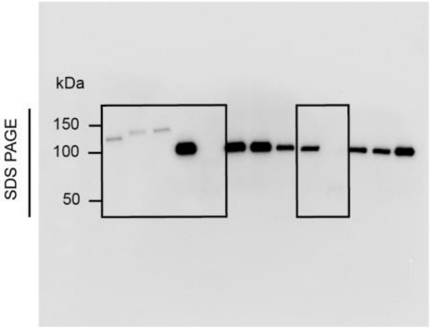

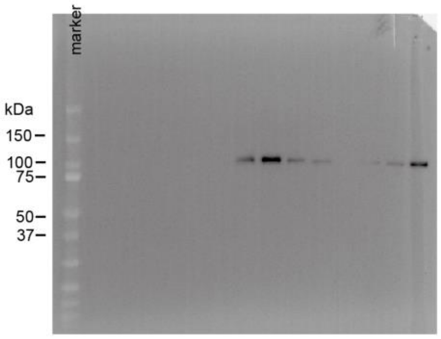

uncropped western blots for Supplementary Fig. 1C - Replicate 2

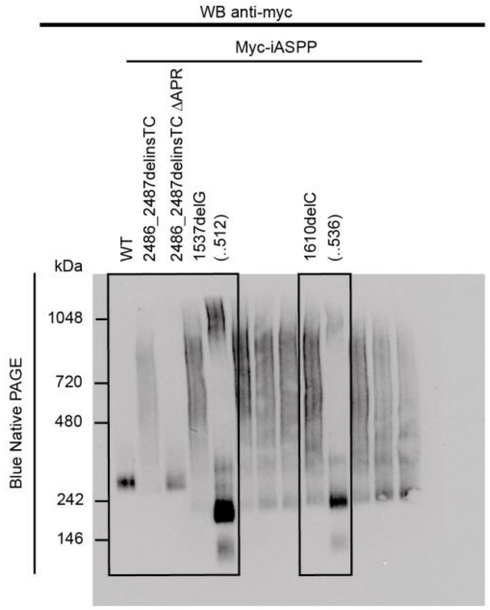

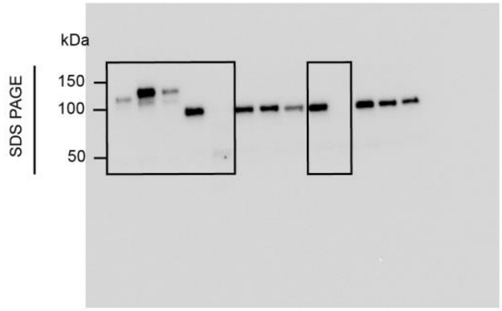

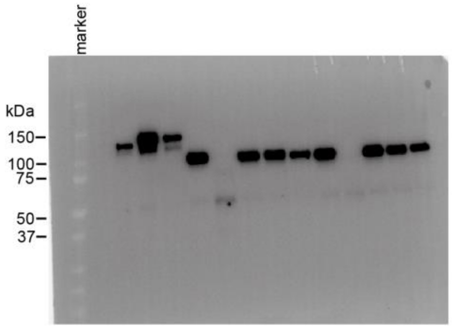

uncropped western blots for Fig. 3B - Replicate 1 (shown in figure)

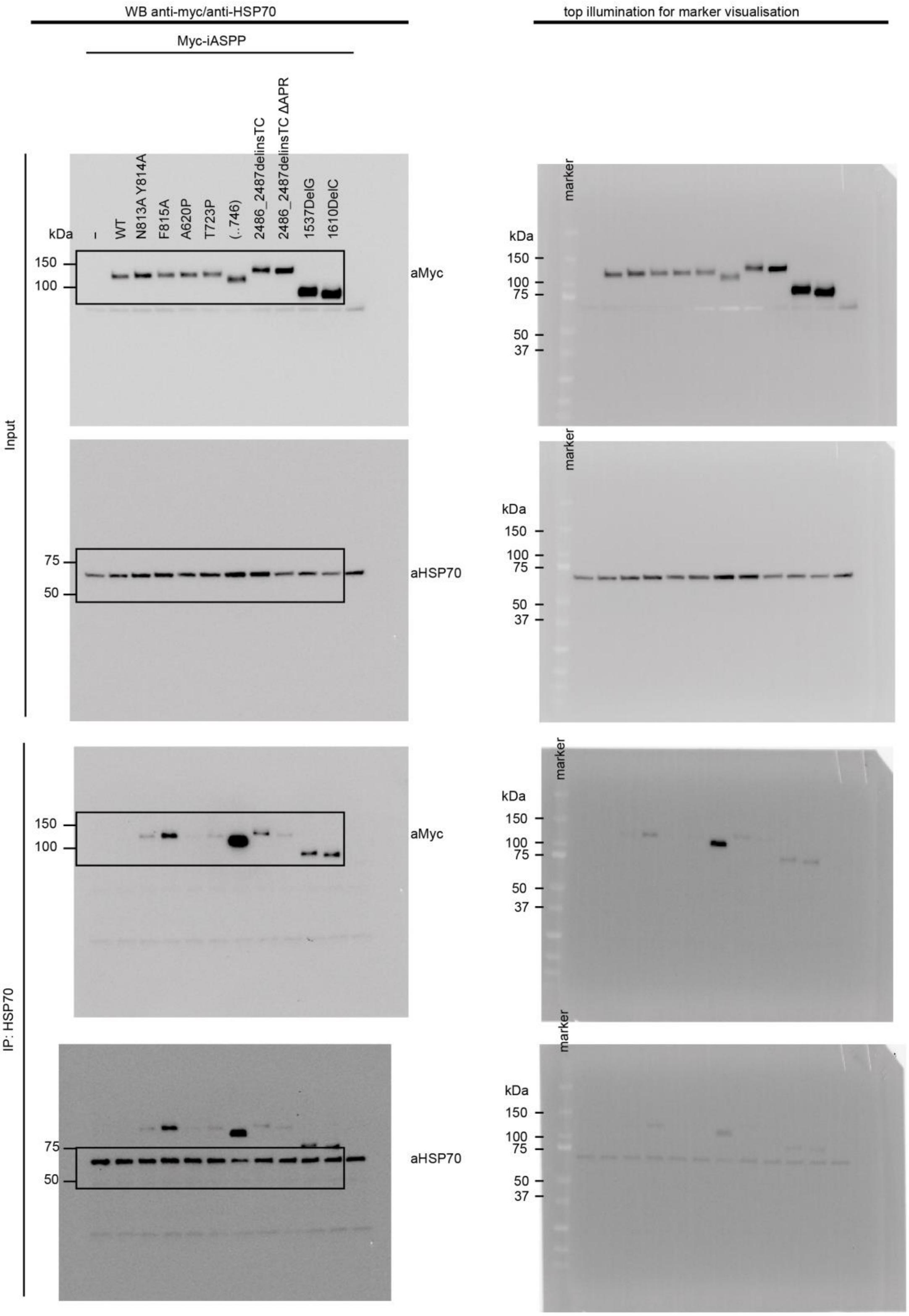

uncropped western blots for Fig. 3B - Replicate 2

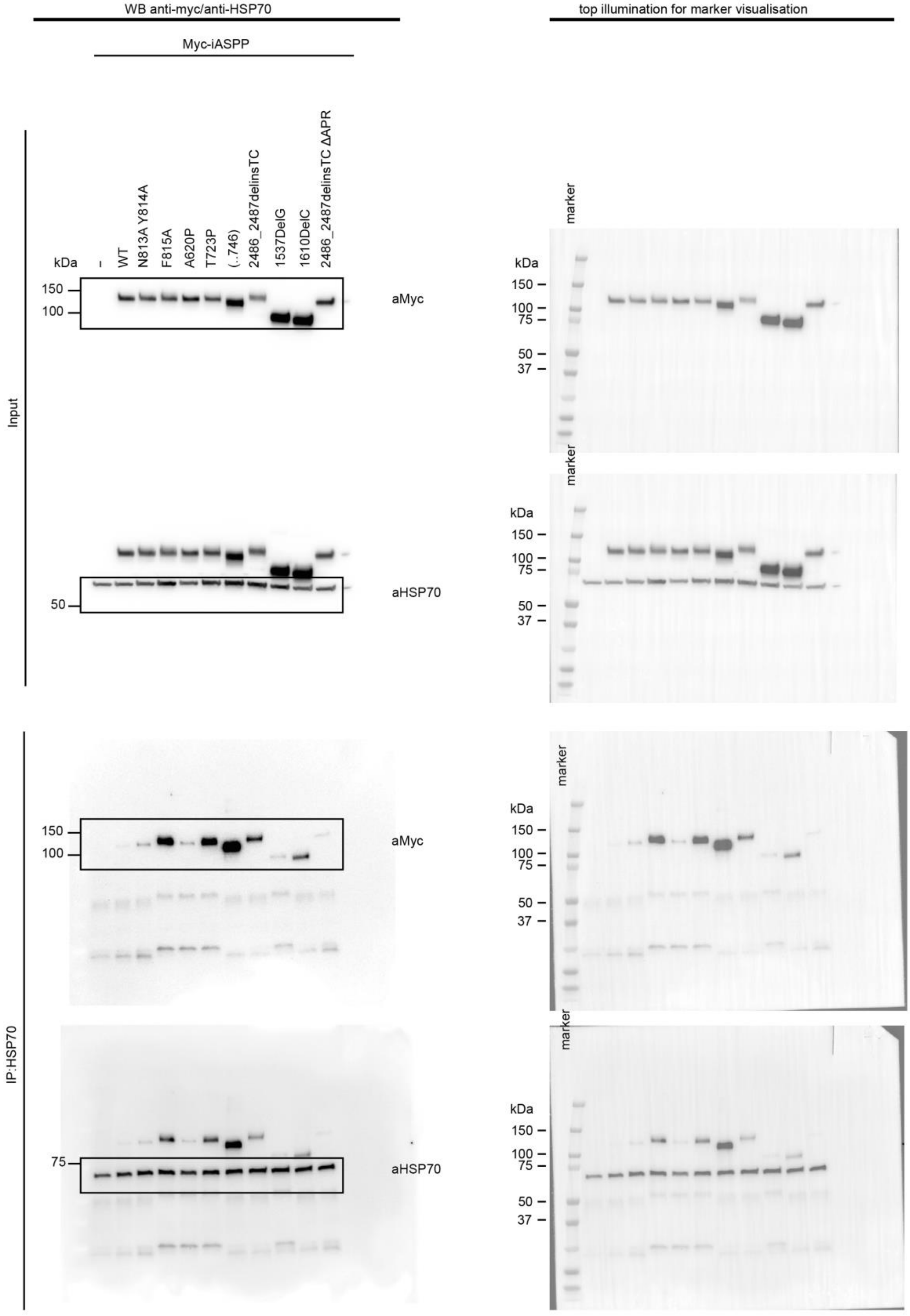

uncropped western blots for Fig. 3B - Replicate 3

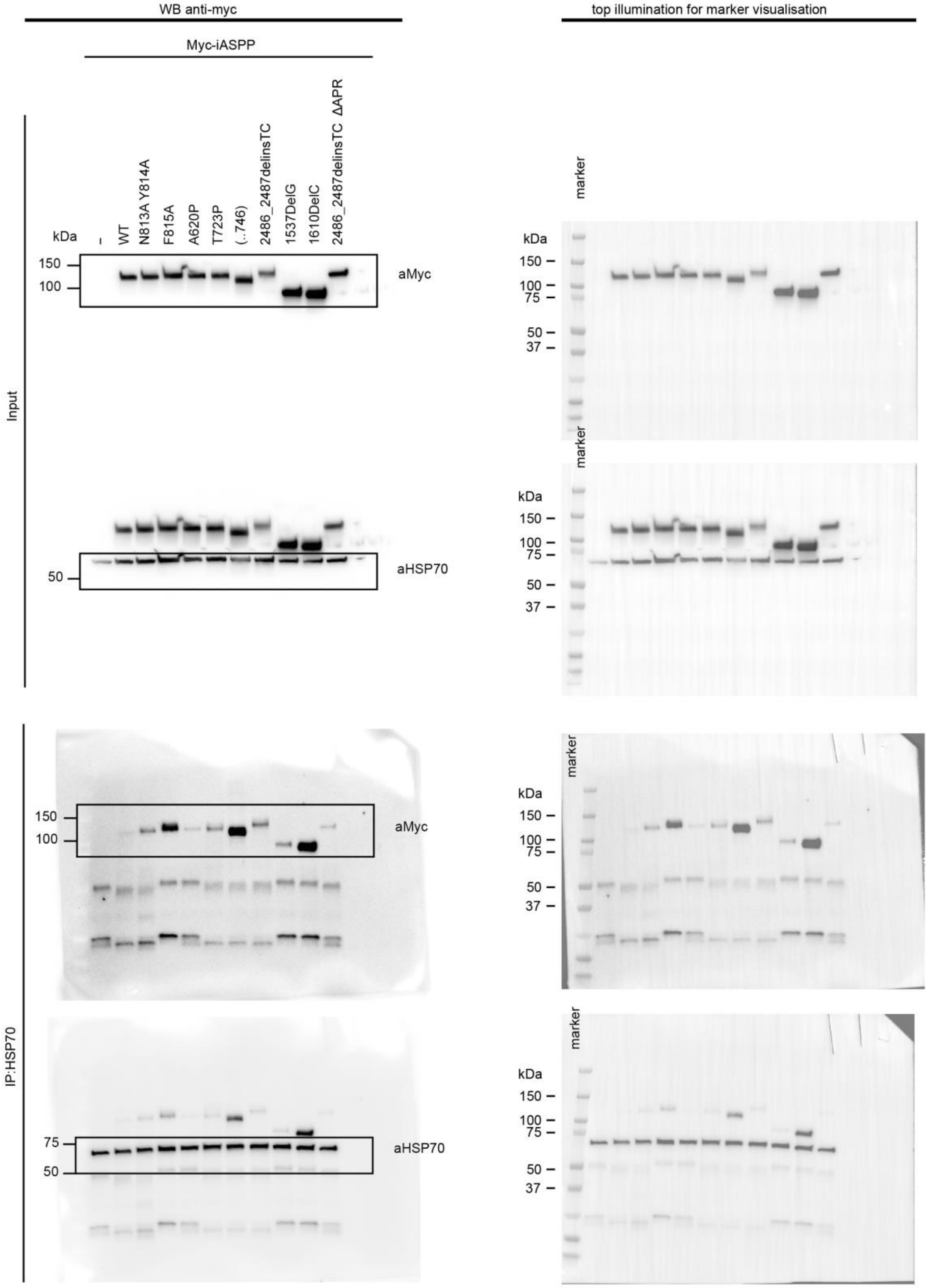

uncropped western blots for Supplementary Fig. 3A

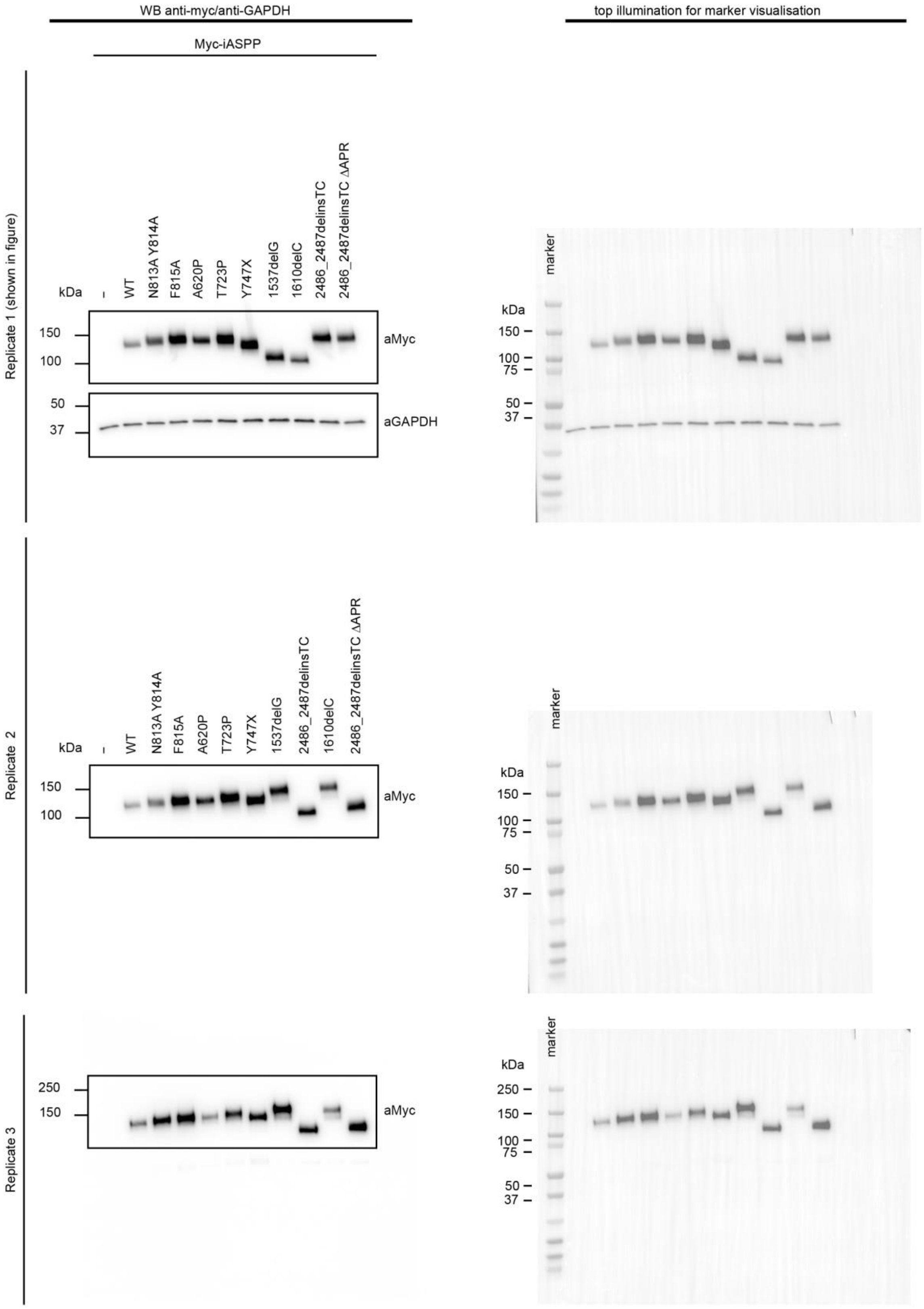

uncropped western blots for Supplementary Fig. 3B - Replicate 1 (shown in figure)

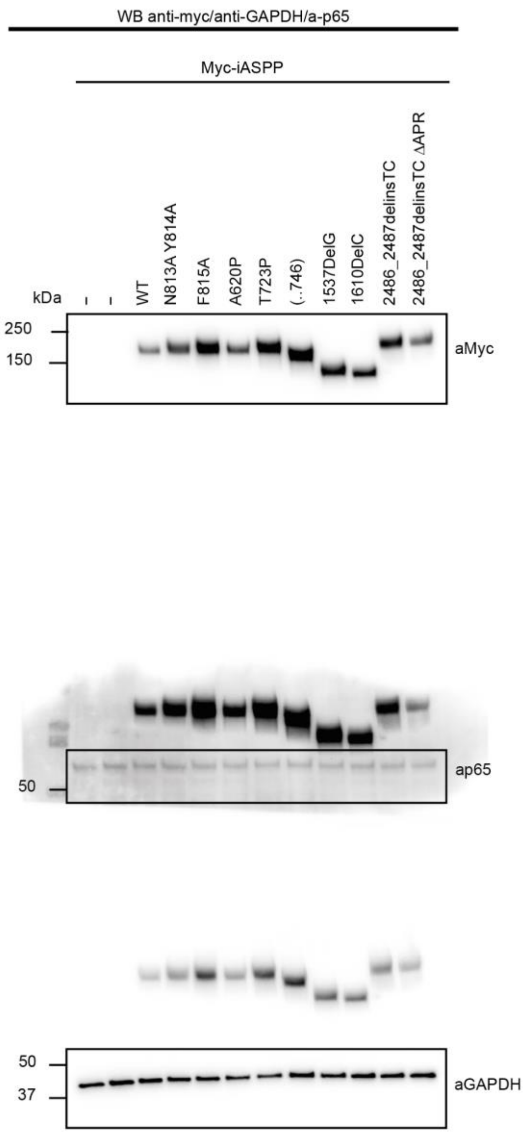

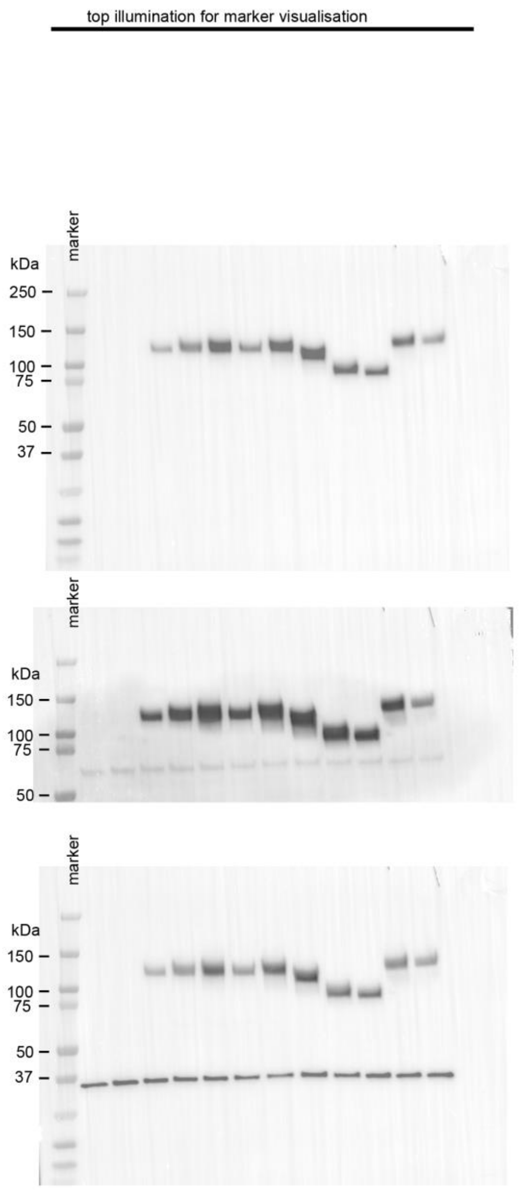

uncropped western blots for Supplementary Fig. 3B - Replicate 2+3

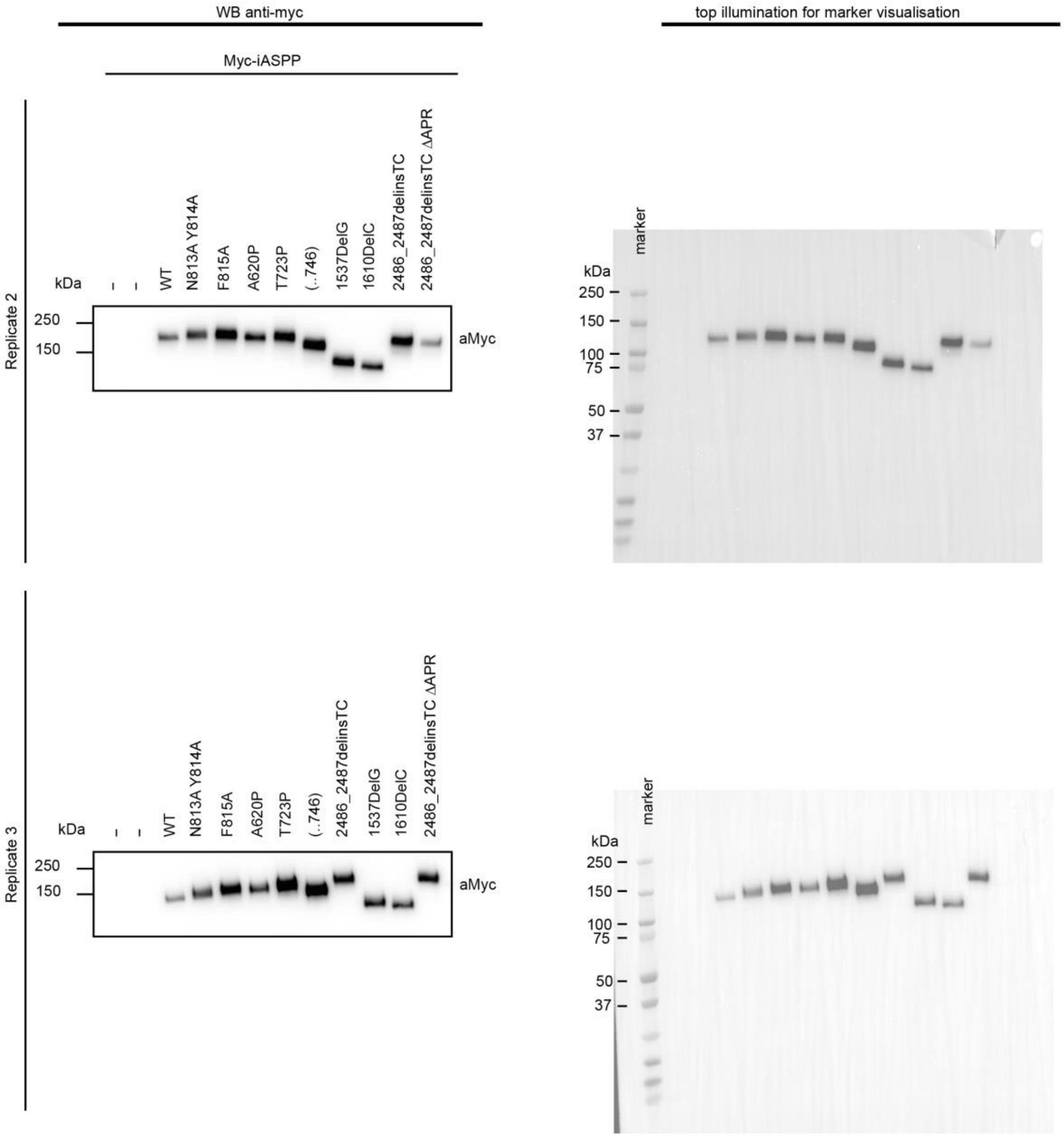

uncropped western blots for Fig. 4B - Replicate 1 (shown in figure)

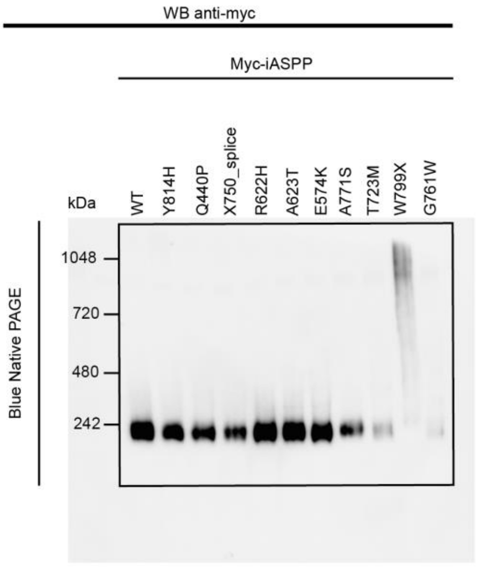

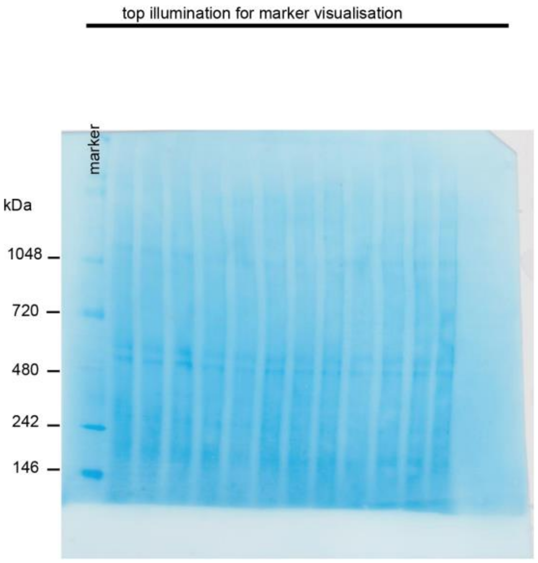

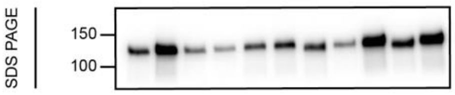

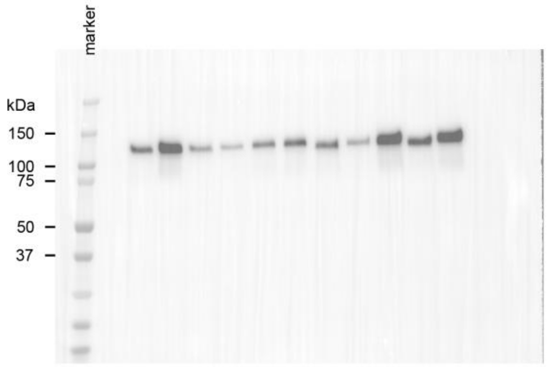

uncropped western blots for Fig. 4B - Replicate 2

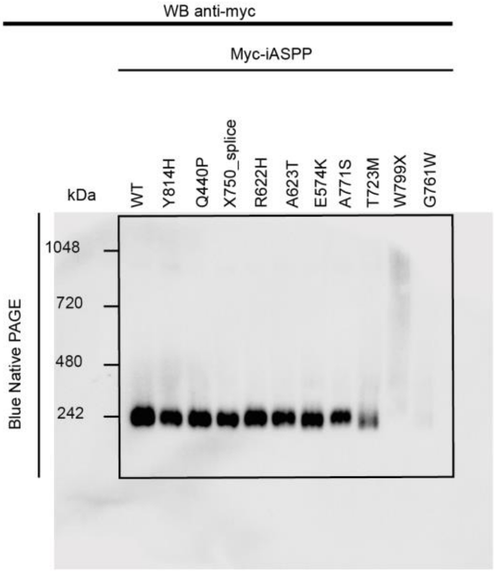

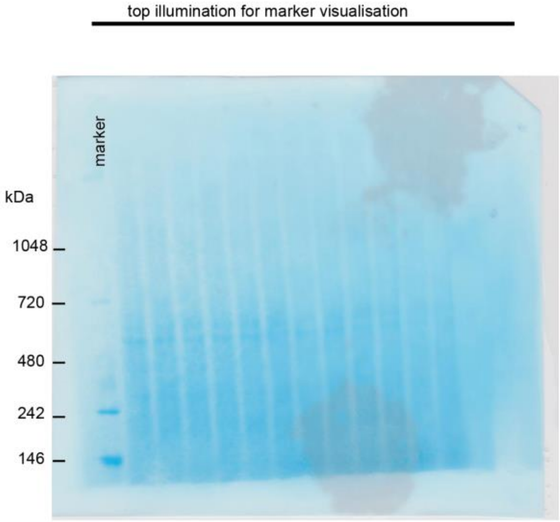

Supplement: Supplementary file 2 — Supplemental Figure 2 [file 41420_2024_2265_MOESM2_ESM.pdf]
